# Enhancing Older Drivers’ Safety: On Effects Induced by Stereotype Threat to Older Adults’ Driving Performance, Working Memory and Self-Regulation

**DOI:** 10.3390/geriatrics1030020

**Published:** 2016-08-24

**Authors:** Lisa Brelet, Ladislav Moták, Magali Ginet, Nathalie Huet, Marie Izaute, Catherine Gabaude

**Affiliations:** 1 Laboratory of Social and Cognitive Psychology (LAPSCO—UMR 6024), CNRS, University of Clermont Auvergne, 63000 Clermont-Ferrand, France; lisa.brelet@univ-bpclermont.fr (L.B.); magali.ginet@univ-bpclermont.fr (M.G.); marie.izaute@univ-bpclermont.fr (M.I.); 2 Unité Mixte de Recherche 5263—CLLE-LTC, CNRS, Université Toulouse 2 Jean Jaurès, Maison de la Recherche, 31100 Toulouse, France; huet@univ-tlse2.fr; 3 Université de Lyon, F-69622 Lyon, France; catherine.gabaude@ifsttar.fr; 4 Laboratory Ergonomics and Cognitive Sciences applied to Transport (LESCOT), The French Institute of Science and Technology for Transport, Development and Networks (IFSTTAR), F-69675 Bron, France

**Keywords:** older, driver, self-regulation, stereotype threat, working memory

## Abstract

In a study concerned with driving behaviors of older drivers (mean age 70 years) in a driving simulator, our findings indicate that telling older drivers that they are more at risk of accidents because of their age and their driving performance-related decline (i.e., exposing them to a stereotype threat concerning older drivers) severely impairs their self-regulatory skills. Moreover, our results show that this is at least partly due to exhaustion of the executive resources (older drivers under stereotype threat tended to contradict the stereotype of being slow by driving faster), appearing also through working memory overload (older drivers under stereotype threat performed markedly less well in a modular arithmetic task than drivers in the control condition). We thus complete the existing evidence that older drivers’ performance may be affected by socially-grounded factors, suggesting that simply being investigated may be enough to tax many capabilities in older people. We also propose that stereotype threat might be at least a partial explanation for why older drivers sometimes have poorer self-regulation performances after attending rehabilitation programs designed to make older drivers safer ones.

## 1. Introduction

Older drivers are often said to attach great importance to the possibility of driving a car. Among other reasons, this might stem from the fact that driving a car is a way to maintain their daily social activities [1,2]. Moreover, giving up these social activities may be accompanied by severe pathological mood impairments, such as depression [3,4,5]. Given that aging is often associated with a decline in those cognitive functions that are involved in driving [6], the question has arisen whether, and to what extent, older adults are able to adjust their driving behavior according to these changes, that is are able to self-regulate [7,8].

Although not unequivocal, the evidence suggests that there is a great deal of self-regulation among normally-aging drivers. On the one hand, it has repeatedly been suggested that older drivers report that they avoid difficult driving situations (e.g., driving during rush hour), congruently with assessments of their cognitive abilities and their confidence assessments [9,10,11]. For example, it has been argued that older drivers reduce their driving at night and in fog, providing their processing speed decreases [12,13,14], and avoid situations in which they feel less confident, such as driving at night and at night when it is raining [15,16]. In addition, there are behavioral data supporting the assumption that healthy older drivers are able to engage in successful self-regulation. For instance, older drivers appear to: (i) overtake less than their younger counterparts when driving on a multilane highway [17]; (ii) postpone distracting activities when approaching left/right turns [18]; and also (iii) avoid difficult driving situations more than their younger counterparts in simulated driving [19]. Finally, it has been concluded that older drivers exhibit self-regulatory patterns, on the basis of safety-related data indicating that healthy older drivers do not necessarily have an overly-increased risk of accident as compared to other populations of drivers [7].

An independent body of research suggests, however, that cognitive, physical and also self-regulatory performance may suffer when one is exposed to negative stereotypes. Indeed, when negative beliefs shared about a social group are highlighted and applicable, they result, under specific circumstances, in performance decreases in members of the group concerned. This phenomenon, called stereotype threat, has already been demonstrated in various domains (including intellectual performance [20], sports [21], mathematics [22,23], driving [24,25,26] and others [27]) and across different age groups ranging from children [28,29] to older adults [30,31,32].

In the context of driving, older adults have not only been found to be subject to stereotype threat [33], but also, consistently with the stereotype threat literature, older drivers under stereotype threat made more errors than drivers of the control group during a simulated driving task [34]. Along the same lines, [35] found that older drivers in threatening conditions presented longer brake reaction times and longer following distances than drivers who were not threatened. The deleterious effects of stereotype threat are particularly salient in older adults who value or otherwise ascribe some importance to driving (i.e., who strongly identify with the performance domain [34]) and in individuals with diminished working memory resources [35]. This is in accordance with the proposed process model of stereotype threat [36].

**Figure 1:**
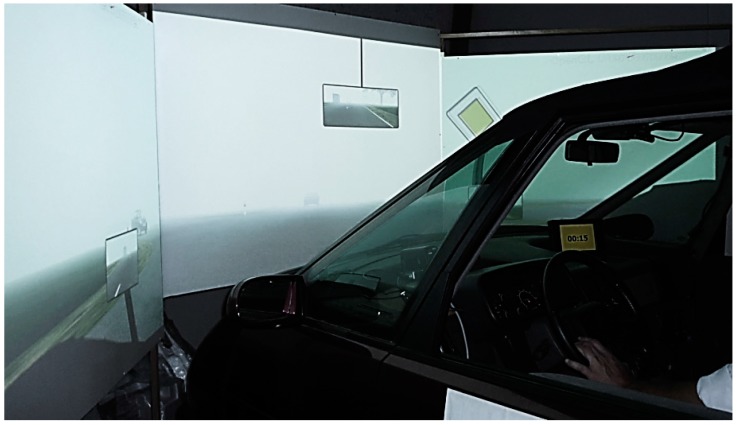
View of the driving simulator during experiments.

Following this model, stereotype threat, that is a threat to one’s integrity, an imbalance between what one expects as success, on the one hand, and the stereotypically-primed performance deemed to be poor, on the other hand, acts as a stressor, leading to physiological stress reactions, cognitive monitoring and interpretative processes, affective responses and efforts to cope with these negative emotions [36]. Whether or not this reaction is more salient in terms of physiological stress response, in terms of monitoring, the self-relevant performance (i.e., to what extent does my performance contradict the stereotyped one?) or in terms of thought suppression processes (i.e., while individuals try to extinguish the tiniest negative affect that would testify to the validity of the stereotype), the domain general resource, as [36] argues (and see also [37,38]), that would always be impacted is working memory (see Figure 1; p. 337, [36]). Therefore, disruption of optimal performance under stereotype threat would commonly occur in a variety of tasks due to working memory overload, despite several concrete pathways and discrete processes that link stereotype threat trigger to this working memory depletion. It is of note that stereotype threat-related negative impacts occur: (i) with an apparent will to contradict the stereotype at hand by whichever means (see [20]); (ii) in individuals who are highly involved in the task described (individuals for whom it “matters”) [20]; and (iii) in tasks that necessitate controlled, in contrast to tasks soliciting automatic, processing [39,40].

Yet, how can information that points to an existing stereotype translate into, or, possibly, hinder, specific self-regulation patterns? In line with both the process model of stereotype threat and the evidence that poorer cognitive performance may stem from the depletion of working memory resources [36,37], it has become obvious to expect that stereotype threat causes a decrease in self-regulation capacity, exactly due to a previous overinvestment of the available resources in contradicting the stereotype at hand [24,41,42]. For example, although attending an elite university, students with low socio-economic status (SES) had more concerns regarding their academic achievements and, consequently, were only able to self-regulate to a lesser extent, that is consumed more food during a task and showed more interference in a Stroop task, than students with high SES [43]. Similarly, Afro-American participants and women under stereotype threat have shown lower performances in tasks demanding some degree of self-control, be they tasks that were related to the stereotype threat at hand (Stroop test for Afro-American students [20]) or that were not (squeezing a handgrip exerciser for women [44]). Finally, this phenomenon has been replicated in women threatened about their lack of mathematical skills and impacts their self-regulation in such various and initially unrelated tasks as aggressive behaviors, eating and decision making [41].

To the best of our knowledge, however, virtually nothing is known regarding the effects of stereotype threat on the self-regulation of older drivers. In this article, we offer some evidence regarding both how stereotype threat impacts older drivers’ self-regulation and the possible causal chain that links stereotyped information to specific patterns of self-regulation. In light of the previously-discussed theoretical frame and empirical evidence [36,37,38,39], the causal chain that we propose and test here is that when told that they are more at risk of accident because of their age and their driving performance-related decline (see [45,46,47] and also [48,49]), older drivers would try their hardest to contradict the stereotype at hand [20,36], which, in turn, would lead to an overinvestment of their working memory resources [37,38,50] and, finally, to the lack thereof for, and a poorer performance in, a subsequent task in which they should self-regulate [41,43,44].

In a previous study in simulated driving settings [19], older drivers drove in four circuits of increasing difficulty (combining, progressively, left turns, high-density traffic and restricted visibility conditions) and were then given a limited amount of time to train for a fictitious driving test. During this self-paced training, older drivers have been found to behave in a strategically-adapted manner given that they concentrated on the two easiest circuits where they certainly might enhance their performance before the oncoming driving test, while younger drivers concentrated rather on the two most difficult circuits. Moreover, both age groups started their training with somewhat easier circuits, moving only later to circuits of greater difficulty, which seems optimal for maximizing their learning uptake and assuring at least some degree of performance, although, even here, the global level of chosen difficulty was again lower for older than for younger drivers. It was therefore concluded that older drivers were as able to self-regulate as their younger counterparts were (see also [51,52]). It might then be expected that within a restricted training time, older drivers under stereotype threat, as compared with their control or even with their younger counterparts, would show no sign of allocating their training time selectively to a specific difficulty of circuits.

In the study described below that follows [19], two groups of older drivers had to complete four increasingly difficult driving circuits in a driving simulator and were then given a limited amount of time to train for a fictitious test. Provided they were able to do so (e.g., [51]), drivers were expected to allocate their training time selectively on the basis of the circuits’ difficulty [51,53]. Before engaging in the initial four circuits, one group of drivers was provided with stereotype threat-inducing instructions. We assumed that under stereotype threat (here, the stereotype of older drivers aged 65 years or over who are more at risk of traffic accidents than younger drivers; see also [33,35]), drivers would no longer be able to self-regulate accurately, that is would not be able to focus on circuits of specific difficulty [41,44] and, rather, would show random patterns of self-regulation, that is they would show random allocation of the available training time to different circuits’ difficulty. We further predicted that this self-regulation impairment would occur: (i) as a consequence of drivers’ conscious efforts to control their behaviors and contradict the stereotype at hand (e.g., drivers would do the contrary of what they had been told, that is drive faster under stereotype threat if they were told older drivers are riskier because of driving slowly [20,40]); and, therefore, (ii) as a consequence of spending working memory resources on tackling this task, that is due to working memory depletion appearing as a worse performance in tasks requiring substantial working memory resources [24,36,38].

## 2. Method

### 2.1. Participants

A total of 67 French older drivers aged 65 years or over took part in the study. All participants scored above 24 on the Mini-Mental State Examination [54] and stated that they were in excellent or good physical and mental health. They further stated that they were not taking any central nervous system medication, had been in possession of their driving license for at least three years and drove between 5 and 25 thousand kilometers each year. Unfortunately, owing to simulation sickness, we were only able to retain the data of 42 participants for further analyses. Drivers were assigned randomly to either the experimental group (i.e., under stereotype threat; *n* = 21) or the control group (i.e., without stereotype threat; *n* = 21; see Table 1). All drivers signed an informed consent form and were treated in accordance with the ethical standards of the American Psychological Association.

### 2.2. Materials

#### 2.2.1. Driving Task Involvement and Manipulation Check

Given that stereotype threat appears only in subjects for whom the task at hand really does matter (see [36] for a review), each driver first had to estimate to what extent “It is important for [him/her] to be a good driver” [37,38]. The stereotype threat manipulation was then checked, at the very end of our design, by the question “If I am not successful in driving the simulator, experimenters will believe that older drivers are less able to drive than younger drivers are”, as drivers under stereotype threat were deemed to be more concerned by the experimenter’s opinion than their control counterparts. Both questions were followed by 9-point Likert scales ranging from 1 (don’t agree at all) to 9 (absolutely agree).

#### 2.2.2. Four Circuits of Increasing Difficulty

Counterbalanced across participants, four circuits lasting 4 min each, that is restricted to exactly 4 min whatever the distance driven by a participant, were presented to participants in a driving simulator. There were at least two reasons for restricting the time and not the distance driven, per participant. First, our aim was to reduce the duration of driving the simulator as this duration was positively correlated with simulator sickness phenomena. Second, and most of all, our measure of self-regulation depends conceptually on the ratio between time spent per circuit (4 min) and time available for training (6 min, that is leading eventually to strategic choices). Allowing drivers to spend more or less time in each of the four circuits would be detrimental to our measure of self-regulation.

The difficulty of the circuits was manipulated in a cumulative manner, by adding one difficulty at each level. For the purposes of our test, we identified and selected: (a) left turns, a typical high-risk situation for older adults [56]; (b) high-density traffic, a situation that older drivers are said to frequently avoid [57]; and (c) driving with restricted visibility (e.g., at night or in fog), also frequently avoided by older drivers [58]. Combining these three difficulties resulted in: (i) the simplest circuit, consisting mainly of straight roads with light traffic of approximately 20 cars; (ii) the second easiest circuit, also consisting only of light traffic, but with four left turns; (iii) a circuit combining the left turns with heavy traffic of approximately 80 cars; (iv) and the fourth and most difficult circuit, where we added foggy weather to Circuit 3 to restrict the drivers’ visibility. All of the driving tasks were performed in a fixed-base fully-equipped driving simulator adapted from an initial Renault Espace cab (Figure 1), including all steering commands and a manual gear. A 3D visualization loop and a traffic simulation model developed by IFSTTAR (using, respectively, “SIM^2^” and “Dr^2^” software) were employed to simulate driving scenarios onto three forward screens with a 150° horizontal and 40° vertical field of view.

#### 2.2.3. Self-Regulation: Perseverance

In order to examine self-regulatory patterns, the drivers were invited to train themselves before a forthcoming (fictitious) test in a self-paced manner, during a restricted time. The allowed training time (6 min) represented about one third of the time needed to drive around all four circuits (4 × 4 min), and drivers were expected to behave in a strategic manner, that is to focus selectively on these circuits where their learning uptake might be optimal, unless they were under stereotype threat and, therefore, with no specific pattern of self-regulation. The variable of interest here was perseverance, that is the proportion of time allocated to each circuit during the self-paced training [51,52]. Zero seconds were allocated to non-selected circuits. All of the instructions were presented in the participants’ native language, that is in French. Translations are provided for the purpose of this paper, and the original French version is available upon request from the corresponding author. The exact wording introducing this training phase was as follows:

“In the previous circuits, your driving was more or less confident, more or less safe. We are now going to allow you a 6-min practice session, where you can train yourself to drive as safely as possible for the next test. Hence, you can go back over exactly the same circuits. Of course, you are free regarding the choice of circuits, the order in which you practice them, and the time you spend on each of them, but you must respect the 6-min time limit. Inside the simulator, on your right, you can see a timer indicating the amount of time left before the end of your training session. If you wish to change from one circuit to another at any time, just say ‘stop’ and announce the number of the circuit you would like to drive around next. What is the number of the circuit you would like to begin with?”

#### 2.2.4. Working Memory Test: Modular Arithmetic

In order to quantify the cognitive resources possibly depleted under stereotype threat, we used a modular arithmetic task (Gauss, 1801, in [38]). Here, participants had to estimate whether the result of specific chains, e.g., 47 = 19 (mod 4) was an integer (true) or a real number (false). To do so, the second number must first be subtracted from the first (i.e., 47-19), and then, this difference must be divided by the last number (i.e., 28/4). Importantly, the difficulty of these operations, and therefore, the working memory demands, can be easily manipulated. This is important because stereotype threat is not easy to capture in easy tasks [38,40], and one might therefore expect an interaction between the difficulty of items and stereotype threat manipulation: in fact, while no difference should occur in easy items, drivers under stereotype threat should show poorer performance as regards difficult items where the working memory resources allocated to tackling the stereotype at hand would appear as detrimental (i.e., causing an overload) to the mathematical problem resolution [36,39,40]. The final task thus consisted of eight easy, e.g., 6 = 3 (mod 2), and eight difficult items, e.g., 47 = 19 (mod 4), including also eight medium (filler) items, e.g., 19 = 3 (mod 4). Following the previous findings [38], we were only interested in the percentage of correct answers at both the easy and the hard level of difficulty and not in resolution times. The task was performed using the DMASTR software (“DMDX”) and an arranged keyboard with buttons “true”/“false” plugged into a personal computer with a 21-inch screen. The authors wish to thank K.I. Forster and J.C. Forster of the University of Arizona for their software, which is freely available [59].

### 2.3. Procedure

Upon their arrival, the participants were presented with a cover story (see [44] for similar examples), and throughout the experiment, we used a pre-recorded video, so as to avoid any experimenter-related effect. The exact wording was as follows:

“Today, we are interested in simulated driving. You might know that driving simulators are tools frequently used when assessing driving safety-related concerns. However, little is known about the impact of these tools on drivers’ perception compared with real driving. This is why today we are trying to assess which changes should be made to the driving simulator tool so as to produce the same psychological processing as if we were driving a real car.”

After this introductory part, all drivers answered a set of socio-demographic questions, including a driving task involvement scale, and were familiarized with the tasks to be done, i.e., driving the simulator (simple training session of 7–10 min) and performing the working memory task, that is modular arithmetic (12 items, 4 of each difficulty). As in previous research bearing on this task [38], only the participants who performed well (100%) or quite well (75%) on easy items were selected for further analyses, in order not to confound the effect of stereotype threat with learning difficulties regarding the modular arithmetic task.

The experimental phase began with the baseline measure of modular arithmetic (24 items, 8 of each difficulty), which occurred right before the stereotype threat priming. In fact, before completing the four circuits of increasing difficulty, drivers of the experimental group heard the following introduction deemed to induce the stereotype threat (adapted from [20,37]). Please note that the introduction ends by specifying in which respects older drivers might be considered to be more at risk of accident, thus prompting older drivers to contradict the stereotype at hand precisely in these aspects:

“You might be aware that people aged 65 years or over drive less safely than younger drivers. Clearly, reports concerned with driving safety emphasize that older drivers have more traffic accidents than younger drivers do. Given that we are not able to explain this phenomenon, the study in which you agreed to participate is designed to help understand these age-related differences. Therefore, your driving performance is going to be compared with the driving performance of other participants, including younger drivers. Indeed, drivers aged 65 years or over are deemed to have more traffic accidents for at least the following two reasons: they drive too slowly and also hesitate too much before taking left turns.”

Drivers of the control group were simply provided with descriptions of the four circuits, and all of the drivers completed the four circuits of increasing difficulty. Afterwards, they were told that they could train for a forthcoming test. During this self-paced training session, we recorded their perseverance. Finally, all of the drivers performed the post-test modular arithmetic task (once again, 8 items of each difficulty), answered several questionnaires concerned with other issues published elsewhere [8], completed the manipulation check and were thanked and debriefed.

### 2.4. Statistical Treatments, Dependent Variables and Design

All dependent variables were treated using the comparison of means, be it through an independent- or a one-sample Student’s *t*-test or through analysis of variance (ANOVA). The size effect is indicated using Cohen’s *d* and partial *η*^2^, respectively. Any effects where *α* < 0.05 were considered significant, but marginally significant effects are also discussed (0.10 > *α* ≥ 0.05).

Driving task involvement and manipulation check variables were submitted to *t*-tests, distinguishing the stereotype threat and the control groups. While driving involvement was expected to show no difference across groups, drivers of the stereotype threat condition were hypothesized to show higher levels of manipulation check, that is higher levels of concern that their driving performance would allow the experimenter to ascertain whether the stereotype at hand is a valid one or not.

Driving performance, used as a proxy of drivers’ determination to contradict the stereotype at hand, was handled in two ways. First, a mixed two-way ANOVA compared the distances (and therefore, the average speed) driven by both groups of drivers across all four circuits as a whole. Second, average speed in specific portions of those four circuits, and mainly in three 90-km/h portions (Circuits 1, 2 and 3) and in three left turns (Circuits 2, 3 and 4), were compared across groups using *t*-tests, in order to specify whether older drivers under stereotype threat adopted higher speeds in the portions of interest. Only these portions were passed through by all drivers.

The perseverance in self-paced training, that is the variable that was crucial in ascertaining the self-regulation patterns of our drivers, was submitted to a two-way mixed ANOVA comparing time spent in different circuits across both groups of drivers. Given the lack of any significant effect between the groups, which was of primary interest in our study, an additional inter-subject variable of drivers’ age category was added, in order to unravel its potential confounding effect on drivers’ perseverance (see below).

Finally, the performance in modular arithmetic was examined through a three-way mixed ANOVA where mean scores were compared across both groups, between easy and hard items and also between baseline and post-test measures. Given that stereotype threat effects should only appear after stereotype threat priming (i.e., post-test) and in hard items, we expected a three-way interaction to appear.

## 3. Results

### 3.1. Driving Task Involvement and Manipulation Check

As can be seen in Table 1 (see above), descriptive values of the driving task involvement question were almost identical in the stereotype threat and control groups, and the very slight difference was not significant. Moreover, whatever the group, the mean scores were significantly greater than the theoretical middle of the scale, *t*(20) = 6.13 and *t*(20) = 10.16 for both, experimental and control groups, respectively, both *p* < 0.001, both Cohen’s *d* > 1.33, indicating that drivers across both groups were involved in driving.

As regards the manipulation check, it appears that our priming was successful in that drivers induced with stereotype threat believed more strongly than their control counterparts that their driving performance would be seen, by the experimenter, as diagnostic of their (stereotyped) group difficulties (Table 1). This effect, by the way, was of considerable magnitude, as Cohen’s *d* = 0.88.

### 3.2. Driving Performance: Distances Driven in the Four Circuits

In the 42 participants selected for our analyses, there was a considerable range regarding the distances that were actually driven by each of the participants. Table 2 summarizes these distances across both groups.

Overall, a 2 (group: stereotype threat vs. control) × 4 (circuit difficulty: 1–4) mixed ANOVA with repeated measures on the second variable and with total distance driven per circuit as the dependent variable tended to show longer distances (and thus, greater speeds within the given time limit) in drivers of the experimental group (mean (M) = 2.97 km, standard deviation (SD) = 0.60 km) than in drivers of the control group, M = 2.67 km, SD = 0.55 km, *F*(1, 40) = 3.86, *p* = 0.06. This effect was of moderate magnitude (*η*^2^ < 0.09), indicating that a fully significant, and not only marginal, effect of stereotype threat might have been achieved had greater sample sizes been available. This ANOVA also yielded a circuit effect, *F*(3, 120) = 113.40, *p* < 0.001, *η*^2^ = 0.74, such that distances in Circuits 1 and 2 were greater than the distances in Circuits 3 and 4, all *p* < 0.001 (Tukey’s HSD test). Finally, there was no significant interaction effect of the two factors, *F*(3, 120) < 1.00, non-significant (*ns*).

### 3.3. Driving Performance: Driving Speeds in Specific Portions

From the situations that were driven through by all drivers, we kept for our analyses three straight lines limited to 90 km/h (Circuits 1, 2 and 3) and three left turns (Circuits 2, 3 and 4), situations that clearly corresponded to the points we stressed in our stereotype threat priming (i.e., pointing out that older drivers were slower on straight lines and hesitated before turning left). A series of Student’s *t-*tests with group (stereotype threat vs. control) as a between-subject factor performed on all three 90 km/h items tended to show significant differences in the predicted directions, that is drivers under stereotype threat adopted marginally higher speeds than drivers in the control condition, all *t*(40) ≥ 1.44, all *p* ≤ 0.08, all Cohen’s *d* ≥ 1.46 (Table 3). These differences, pointing toward the same pattern, were even more clearly pronounced in the case of left turns in Circuits 2 and 3, both *t*(40) ≥ 1.85, both *p* ≤ 0.04 and both Cohen’s *d* ≥ 0.57. This difference remained marginal, again, in the case of the left turn in Circuit 4, *t*(40) = 1.31, *p* = 0.09, *d* = 0.40. Although several of the observed differences did not reach the predicted significance threshold of *α* < 0.05, the observed results, and the associated size effects, tend to go in the direction of our hypothesis that drivers under stereotype threat engaged in contradicting the stereotype at hand and mainly in these specific portions that were directly targeted by our priming.

### 3.4. Self-Regulation: Perseverance

Contrary to our expectations, a 2 (group: stereotype threat vs. control) × 4 (circuit difficulty: 1–4) mixed ANOVA only revealed a marginal effect of circuit, *F*(3, 120) = 2.32, *p* = 0.08, *η*^2^ = 0.06, indicating that drivers were not insensible to the circuit’s difficulty. However, neither the effect of group nor the group × circuit interaction appeared as significant, both *F* < 1.00, *ns*.

Given that samples of older adults may easily appear heterogeneous in self-regulatory patterns (e.g., [60]), it is recommended that these samples be further distinguished into smaller and more coherent age subgroups. In this vein, and in order to unravel the potentially confounding effect of age, we divided both the experimental and control groups into subgroups of younger-older drivers (n_1_ = 11, M = 67.64 years, SD = 1.43, and n_2_ = 15, M = 68.20 years, SD = 1.74, for groups under vs. without stereotype threat, respectively) and older-older drivers (n_3_ = 10, M = 74.40 years, SD = 4.74, and n_4_ = 6, M = 75.33, SD = 4.84, respectively). This distinction was made on the basis of mean drivers’ age (70 years), and younger-older drivers are thus represented by all drivers up to 70 years and older-older drivers by all drivers aged 71+ years. Drivers’ self-regulatory patterns were then investigated applying a 2 (group: stereotype threat vs. control; between-subject factor) × 2 (age: younger-older vs. older-older; between-subject factor) × 4 (circuit difficulty: 1–4; within-subject variable) mixed ANOVA. This procedure revealed the main effect of circuit, *F*(3, 114) = 3.81, *p* = 0.01, *η*^2^ = 0.09, and circuit × age interaction, *F*(3, 114) = 4.09, *p* = 0.01, *η*^2^ = 0.10. Most importantly, however, this ANOVA yielded the expected three-way interaction, *F*(3, 114) = 3.18, *p* = 0.03, *η*^2^ = 0.08.

**Figure 2:**
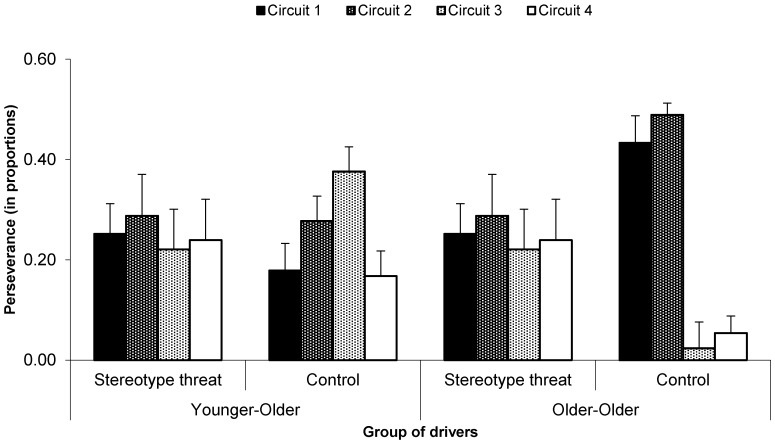
Mean proportion of time (perseverance) allocated to driving circuits of different difficulties and following stereotype threat priming and distinction of driver ages. Error bars indicate the standard error of the mean.

As can be seen in Figure 2, when under stereotype threat, there was no difference in the time allowed for various circuits (that is, in perseverance), be it following the driver’s age or the circuit’s difficulty, all post hoc *p* ≥ 0.64. On the other hand, very adaptive self-regulatory patterns did appear in the control condition. Here, younger-older drivers without stereotype threat focused their self-paced training mainly on Circuit 3 and less on Circuits 1 and 4, both *p* ≤ 0.05. Older-older drivers focused their self-paced training mainly on Circuits 1 and 2 and less on Circuits 3 and 4, all *p* ≤ 0.001. This suggests that while drivers without stereotype threat were able to target a specific difficulty level that might be a proxy of their adapted self-regulation, this was not the case for drivers under stereotype threat, who did not target any circuit difficulty in particular and showed a rather random pattern of self-regulation.

It is further of note that within control groups, older-older drivers focused their self-paced training more than younger-older drivers on Circuits 1 and 2, and less than younger-older drivers on Circuit 3; all *p* ≤ 0.02. However, no such difference was observed within experimental groups, all *p* ≥ 0.57. This indicates that the drivers not submitted to stereotype threat showed not only difficulty-specific, but also age-specific patterns of self-regulation. In fact, the older the drivers, the easier the preferentially-chosen circuits. However, here again, no such age-related adjustment could be seen in drivers under stereotype threat, suggesting that stereotype threat hindered self-regulation.

### 3.5. Working Memory Test: Modular Arithmetic

A 2 (group: stereotype threat vs. control) × 2 (item difficulty: easy vs. hard) × 2 (measure time: before vs. after stereotype threat priming) mixed ANOVA with repeated measures on item difficulty and measure time and with the percentage of correct answers as dependent variable revealed two main effects, *F*(1, 18) = 6.47, *p* = 0.02, *η*^2^ = 0.26 for group and *F*(1, 18) = 38.16, *p* < 0.001, *η*^2^ = 0.68 for item difficulty, but also several interactions. First, there was a group × item difficulty two-way interaction, *F*(1, 18) = 4.99, *p* = 0.04, *η*^2^ = 0.22, and also an item difficulty × measure time two-way interaction, *F*(1, 18) = 6.83, *p* = 0.02, *η*^2^ = 0.28. Most of all, however, this ANOVA yielded the expected, although only marginal, three-way interaction, *F*(1, 18) = 3.99, *p* = 0.06, *η*^2^ = 0.18.

**Figure 3:**
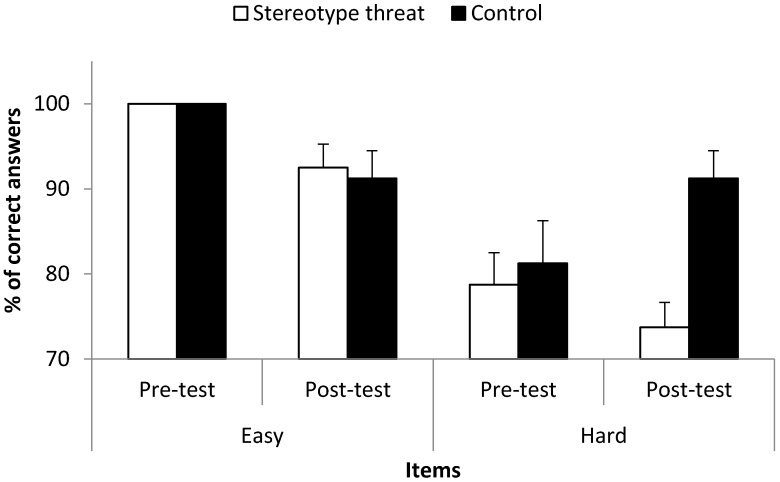
Mean percentage of correct answers across groups, difficulty of items and different measurement times. Error bars indicate the standard error of the mean.

Although seemingly apparent from Figure 3, there was no difference between the two groups in pre- vs. post-test regarding the easy items, *p* = 0.77. However, while the two groups did not differ regarding the difficult items in pre-test (*p* = 0.70), they did so in post-test (*p* = 0.001), the mean difference being almost 20% of correct answers, Cohen’s *d* = 1.79. Put differently, while the control group drivers tended to enhance their performance in hard items from pre- to post-test (*p* = 0.06), no such enhancement was observable in drivers under stereotype threat (*p* = 0.19), suggesting that tackling the stereotype threat at hand was working memory resources consuming.

## 4. Discussion

Our findings suggest that exposing older drivers to information on their possible age-related decline and stressing how this might increase their risk of accidents, leads them to self-regulate in a much less strategic way than control drivers are capable of. This result, while being somewhat counterintuitive at first glance, is fully in line with predictions from stereotype threat theory [36,39], which predicts that the threatened subjects are supposed to: (i) engage in conscious control of their behavior [20,40]; (ii) spend a substantial part of their working memory resources on tackling this task [37,50]; and (iii) lack exactly these resources in any subsequent task that would further imply their use, ranging from pure working memory tasks [38] through attentional allocation to other daily tasks, such as driving [24], and up to tasks requiring some degree of attentional or even physical self-control [41,44].

Our study lends at least some initial support to all of these predictions. Those older adults who were told they were more at risk of accidents in specific driving situations, such as straight lines and left turns, tended, on average, to drive faster than their control counterparts, exactly in these situations, but also in all four circuits in general. Interestingly, this is in line with findings of [34] where older drivers under stereotype threat committed more speeding infractions, as well; although in this case, they were simply primed with information that the experimenters’ intention was to investigate why adults aged 65 or above were more involved in on-road accidents, without further specifying which situations would be of particular concern [34]. In fact, empirical evidence suggests that “older driver” stereotypes typically endorse slower driving speeds and left turn-related difficulties [33], and the increased speed of our older drivers under stereotype threat in the four circuits as a whole also seems to underpin this idea. In sum, however, older drivers under stereotype threat in our study at least tended to contradict the stereotype at hand in a more or less systematic manner.

This effort to contradict, however, had its costs. Unlike those from the control condition and although in a task that was otherwise unrelated to driving (i.e., modular arithmetic [38]), the older drivers under stereotype threat suffered from having allocated extra working memory resources to the driving task. While there was no significant difference regarding the easy items, older drivers under stereotype threat had a lower success rate in dealing with hard items than had their control counterparts, suggesting that part of their working memory resources was dedicated to managing the stereotype threat and, as our data tend to suggest, to contradict it (see also [24]). Thus, in line with the process model of stereotype threat [36], we observed that stereotype threat taxed working memory resources [37], although more research is needed to ascertain which concrete pathways (e.g., particular emotional settings, such as anger [61] or anxiety [27], including their suppression, [50]) led to this effect. As [36] puts it, whether the first reaction to stereotype threat consists of a physiological stress response, of self-relevant performance monitoring or of negative thoughts and their suppression, the core cognitive component that is commonly impacted is the working memory, and this also was the case in our study in our older drivers.

The most important finding, still, is that stereotype threat seems to hinder the older drivers’ ability to self-regulate in a strategic manner. Our data on drivers that were not impacted by stereotype threat suggest, in accordance with [19], that drivers of different ages are quite able to self-regulate in a strategic manner as they take into account both their age and the circuit’s difficulty during self-paced training, that is they are able to focus on specific items where their learning uptake presumably approaches optimal values [51,52,53,62,63]. This is, by the way, in line with studies indicating that older drivers do reasonably regulate their driving in other driving tasks, such as overtaking [17] or postponing distracting activities when approaching intersections [18], and is also congruent with the research line concerned with older drivers’ self-declared driving avoidance and its link to their cognitive abilities ([8,10,64], although see [65]).

Under stereotype threat, however, older drivers exhibited rather random patterns of self-regulation, that is patterns that did not appear to be adjusted either to their age or to the circuit’s difficulty. On the one hand, stereotype threat has been shown to diminish self-regulatory and self-control skills in various tasks [41,43,44], and this is what we observed in our study. In addition, it has been argued that self-regulatory skills depend on the available working memory resources [66,67], and this was the case in our study, as well. Our findings thus complement the existing body of research on stereotype threat by confirming that its predictions apply not only to older drivers’ driving performance, but also to older drivers’ self-regulation. An interesting future research avenue would consist of exploring whether such effects remain even as regards other driving variables associated with self-regulation, and such research might concern both self-declared, as well as behavioral variables. Moreover, a comparison of driving simulator vs. real driving data could be of importance.

**Figure A1:**
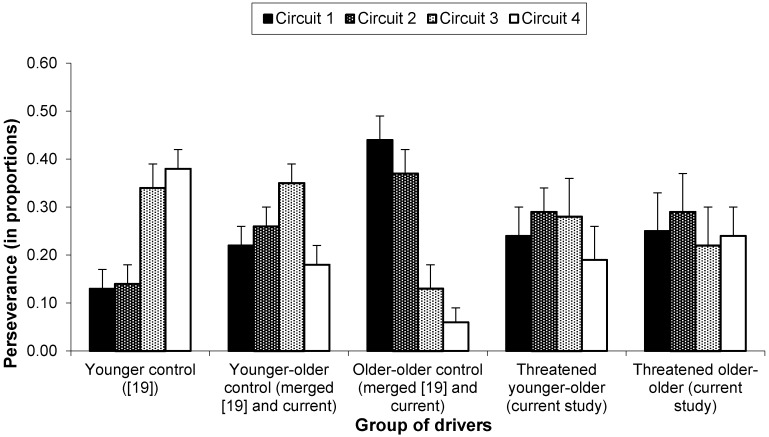
Mean proportion of time (perseverance) allocated by different groups of drivers to different circuits in the self-paced training. Error bars indicate the standard error of the mean. Comments: Younger drivers from the 2012 study [19] spent more of their training time in Circuits 3 and 4 than in Circuits 1 and 2, all *p* < 0.006. No difference was observed either between times spent in Circuits 3 and 4 (*p* = 0.99) or between times spent in Circuits 1 and 2 (*p* = 0.88). A focus on slightly easier items was observed in the younger-older control drivers, as they spent less of their time in Circuit 4 than younger drivers did (*p* < 0.01) and also more of their training time in Circuit 3 than in Circuit 4 (*p* = 0.01) and marginally more of their training time in Circuit 3 than in Circuit 1 (*p* = 0.09). Finally, the older-older control drivers focused their training time mainly on Circuits 1 and 2 and reliably less on Circuits 3 and 4, all *p* < 0.003, while there was no difference between times spent in Circuits 1 and 2 (*p* = 0.75) and in Circuits 3 and 4 (*p* = 0.63). This self-regulatory pattern was totally inverted in comparison with the one exhibited by younger drivers from the 2012 study (all *p* < 0.03), and the older-older drivers also spent more time in Circuit 1 and less time in Circuit 3 than the younger-older control drivers did (both *p* = 0.02).

Beyond the described study, further preliminary support of our main finding on the deleterious effect of stereotype threat on older drivers’ self-regulation comes from [19]. We merged the perseverance-related data from the current study and from [19] and performed additional analyses on the whole pool of data. This was possible given that the experimental design was exactly the same, except for the stereotype threat priming. As can be seen from Table A1 (see Appendix A), merging those two studies resulted in five subsamples of drivers ranging in age from 30 to more than 75 years and in much more statistically-acceptable sample sizes, mainly as regards the control groups of younger-older and older-older drivers. A 5 (group of drivers: young vs. younger-older control vs. older-older control vs. threatened younger-older vs. threatened older-older drivers) × 4 (circuit difficulty: 1–4) mixed ANOVA with repeated measures on the second variable and perseverance as the dependent variable revealed the expected group of drivers × circuit difficulty interaction, *F*(12, 222) = 4.69, *p* < 0.001, *η*^2^ = 0.20. As can be seen from Figure A1 (see Appendix A), the most impressive difference consisted of clearly discernible and age-specific allocation of training time in the control groups of any age, on the one hand, and its complete absence in the groups under stereotype threat on the other. More research is certainly needed to deepen our understanding of how stereotype threat acts on older drivers’ self-regulation, but the preliminary data we provide in this article indicate that older drivers might achieve better self-regulation when not threatened by the omnipresent information that they show high accident rates or diminished driving performance.

One of the possible applications that warrants further research regards the way older drivers may be helped to be safer ones. There is evidence that rehabilitation curricula deemed to enhance drivers’ self-regulatory skills do not necessarily lead to the expected results [46,47]. In fact, while many preventive measures have been designed to enhance drivers’ awareness of their own cognitive functioning [49,68], the effectiveness of such curricula in terms of older drivers’ self-regulation has yet to be proven [69]. If anything, some of these curricula proved to impair older drivers’ self-regulation, that is led objectively to an increase in older drivers’ traffic accidents [47], despite very positive results regarding subjective descriptions given by drivers and considered as indicative of enhanced self-regulatory abilities [45]. This counterintuitive effect extends well over several studies and rehabilitation curricula [46], and although it does not necessarily touch all age groups of elderly drivers (the participants concerned in [46] were men aged 75 years and over; in [47], two thirds of the sample were men aged 74 years on average, suggesting some similarities across the two studies), it remains potentially concerning. Regarding the content of such curricula, it appears that many of them tend to provide older drivers with general information about presumable age-related cognitive declines and the associated driving safety-related concerns. Put differently, older drivers are told in which situations they may become more at risk of accidents providing they experience a particular cognitive decline [45,48,70]. Yet, presenting older drivers with such information resembles situations provoking stereotype threat [20,71], which, as [33] shows, does exist. While many questions remain in this regard (e.g., might stereotype threat extend over the long run, say, of several years, e.g., [47], and what would be the possible effects and evolutions over time, e.g., [23]?), we are still convinced that, first, this research niche merits further interest and that, second, while stereotype threat is “in the air” [20], avoiding possible stereotypes every now and then simply matters (e.g., [72,73]).

In this vein, which means could be put forward to overcome possible stereotype threat-related effects? The available literature has identified at least two ways that seem both feasible and efficient [36] and that might presumably apply as well in the population of older drivers. On the one hand, while stereotypes induce a threat by making salient one’s membership of a stereotyped group [71], one way of counteracting their effects is to allow individuals to identify with other, more positively-connotated groups or individuals at the same time. For instance, it is recognized that women’s performance suffers from stereotype threat when the tasks are presented as being diagnostic of women’s mathematical abilities [23]. Yet, drops in performance may be avoided when women: (i) undergo stereotype threat priming (such as indicating that it is of interest to know why women perform worse in mathematics than men); but (ii) also have an opportunity to identify with otherwise valuable self-relevant groups, such as “college students” (which is positive in comparison with “non-college students” [71]) or “participants in psychology experiments” (where women would be better participants than men [74]; see also [75]). Curriculum-based intervention programs thus might benefit not only from introducing older drivers as being concerned by cognitive aging (negative stereotype), but also as presenting those older people who tend to remain active and socially involved (positive stereotype). This would presumably help older drivers to choose the positive, and not the conflicting, negative identity [71], leaving the working memory resources unaffected by a possible stereotype threat.

In addition, it appears that simply reappraising the situation and the possible stereotype threat-related side effects may help individuals to cope with the associated cognitive and emotional outcomes [36]. In the studies of [50], participants restored their working memory resources and increased their performance after having reappraised either the situation (i.e., were invited to observe their task at hand from an objective, and not personal, point of view; [50], Study 2) or their anxiety (i.e., were invited to consider the anxiety resulting from stereotype threat priming not as being harmful, but rather to conceive of it as a helpful way to increase their performance; [50], Study 3). A similar performance-saving effect has been achieved when explaining to women participants the rationale of stereotype threat, which, in turn, helped them to attribute anxiety to stereotype threat mechanisms and led, in fine, to a reduction of stereotype threat-related detrimental effects [76]. In the same vein, older drivers might presumably benefit from being aware not only of their age-related risks in particular driving situations (which might arguably increase the risk of stereotype threat priming), but also of the underlying stereotype threat-related mechanisms and how to reappraise them usefully. Such an approach would presumably lead older drivers to self-regulate better in the long run, despite potential priming of age-related stereotypes.

## 5. Conclusions

One way of approaching the issue of older drivers’ safety is to look at their self-regulation skills. Evidence from the present research suggests that older adults under stereotype threat, that is fearing to confirm the negative stereotype of older driver, not only modify their driving performance but also show random, rather than adapted, patterns of self-regulation. We also propose preliminary evidence that this may occur due to working memory depletion. Future research will contribute to deepen our understanding of concrete pathways that link stereotype threat triggers to specific self-regulation patterns, and will thus pave the way to measures safely promoting better self-regulation in older drivers.

## Figures and Tables

**Table 1:** Mean values and standard deviations of participants’ main characteristics across both the stereotype threat and control samples.

Variable	Stereotype Threat		Control		*t*	*p*	Cohen’s *d*
	*Mean*	*SD*	*Mean*	*SD*			
Male/female ratio	16/5		17/4				
Age (in years)	70.86	4.81	70.24	4.35	0.44	0.66	0.14
Annual mileage (in thousands of km)	14.48	6.36	13.67	6.28	0.42	0.68	0.13
Mini-Mental State (score out of 30)	29.00	0.78	29.19	1.29	0.58	0.57	0.18
Driving task involvement (9-point scale)	8.00	1.84	7.93	1.33	0.14	0.89	0.04
Manipulation check (9-point scale)	4.67	2.58	2.74	2.15	2.84	0.01 *	0.81

Note: *SD*—standard deviation; *t*—value of Student’s *t*-test for two independent samples; *p*—*p*-value; * *p* < 0.05. The Mini-Mental State (range 0–30) is a French translation of the original Mini-Mental State [55]. None of the mentioned differences reached significance across groups, except for the manipulation check.

**Table 2:** Mean, standard deviation, minimum and maximum values of distances driven by the participants across different circuits (in kilometers).

Circuit	Stereotype Threat				Control			
	*Mean*	*SD*	*min*	*max*	*Mean*	*SD*	*min*	*max*
1	3.65	7.58	2.54	5.20	3.27	8.00	1.84	4.55
2	3.39	5.70	2.15	4.49	3.06	4.93	2.02	3.88
3	2.34	6.38	1.51	3.36	2.00	4.25	1.18	2.84
4	2.51	4.42	1.90	3.46	2.35	5.00	1.19	3.05

Note: *SD*—standard deviation; *min*—minimal values; *max*—maximal values.

**Table 3:** Mean values and standard deviations (in kilometers per hour) and Student’s *t*-test statistics of participants’ speeds in specific portions of simulated circuits.

Circuit Portion	Stereotype Threat		Control		*t*	*p*	Cohen’s *d*
	*Mean*	*SD*	*Mean*	*SD*			
90 km/h Circuit 1	67.10	18.18	59.04	15.41	1.55	0.07 #	0.48
90 km/h Circuit 2	61.92	16.34	55.37	12.85	1.44	0.08 #	0.45
90 km/h Circuit 3	57.02	10.33	51.80	12.46	1.48	0.07 #	0.46
Left turn Circuit 2	28.51	5.90	24.62	5.58	2.19	0.02 *	0.68
Left turn Circuit 3	11.56	4.25	9.61	2.74	1.85	0.04 *	0.57
Left turn Circuit 4	23.33	4.64	21.6	3.92	1.31	0.09 #	0.40

Note: *SD*—standard deviation; *t*—value of Student’s *t*-test for two independent samples; *p*—*p*-value; # *p* < 0.10; * *p* < 0.05.

## Appendix A

**Table A1:** Mean values and standard deviations of participants’ ages (in years) and annual mileage (in thousands of kilometers) across different experimental conditions.

Variable	Younger Control		Younger-Older Control		Older-Older Control		Threat. Younger-Older		Threat. Older-Older	
	*Mean*	*SD*	*Mean*	*SD*	*Mean*	*SD*	*Mean*	*SD*	*Mean*	*SD*
*N*	21		23		14		11		10	
M/F ratio	13/8		16/7		12/2		8/3		8/2	
Age	29.67	3.38	68.00	1.76	75.64	4.34	67.64	1.43	74.40	4.74
Annual mileage	15.38	8.06	14.09	8.27	12.93	6.18	15.23	7.05	13.65	5.76

Note: *SD*—standard deviation; Threat. stands for threatened. Age is indicated in years, annual mileage in thousands of kilometers per year.
